# A Two-Month-Old Infant With a Labial Mass: A Case Report

**DOI:** 10.7759/cureus.98117

**Published:** 2025-11-29

**Authors:** Sujung Kim, Kathryn H Pade, Atim U Ekpenyong, Shahfar Khan, Mylinh T Nguyen

**Affiliations:** 1 Department of Pediatrics, Division of Emergency Medicine, University of California San Diego, San Diego, USA

**Keywords:** canal of nuck, infant, labia, ovary, point-of-care ultrasound

## Abstract

Newborns with inguinal hernias typically present with groin or inguinal swelling, with or without pain. In females, ovarian involvement in an inguinal hernia must be considered, as it is associated with a higher risk of incarceration than a hernia containing bowel. Point-of-care ultrasound can be used in the ED for rapid assessment and diagnosis. We report the case of a two-month-old previously healthy female who presented to the ED with three days of a firm mass in the right labia majora. She was otherwise asymptomatic, feeding and stooling well, and had no vomiting. Her physical examination was notable for a soft, mobile, round structure palpable in the right labia majora. Point-of-care ultrasound revealed a herniated ovary within the canal of Nuck. The patient underwent surgical repair, and the viable ovary was returned to the peritoneal cavity.

## Introduction

The prevalence of inguinal hernias in the newborn period is 1-2% [1]. Risk factors for the development of an inguinal hernia include prematurity, constipation, and lung disease or mechanical ventilation, which increase intraabdominal pressure [2-5]. The embryologic development of the inguinal canal begins with the formation of the processus vaginalis, an outpouching of the parietal peritoneum that invaginates into the inguinal canal [2-12]. The processus vaginalis normally closes around the eighth month of fetal development. When it fails to close in females, it is referred to as the canal of Nuck [1-3,5,6,8-17]. An inguinal hernia can develop through this structure, potentially containing pelvic contents such as intestines, ovaries, fallopian tubes, uterus, urinary bladder, fluid, and/or omentum [1-3,6,8,10,12-14,16,18]. Patients typically present with groin or inguinal swelling, with or without pain [2,5,10,12,16].

This case study presents a two-month-old female with a labial mass, found to contain a herniated ovary.

## Case presentation

A two-month-old previously healthy female, born full-term, presented to the ED with three days of a firm mass in the right labia majora. She had otherwise been doing well, without vomiting, diarrhea, or pain. She had good oral intake and urine output and normal bowel movements.

On examination, the patient was well-appearing. She had a soft, nontender abdomen with normal bowel sounds. Genitourinary examination revealed a palpable, round structure in the right labia majora that was soft and mobile. There was no overlying erythema or pain with palpation.

**Figure 1:**
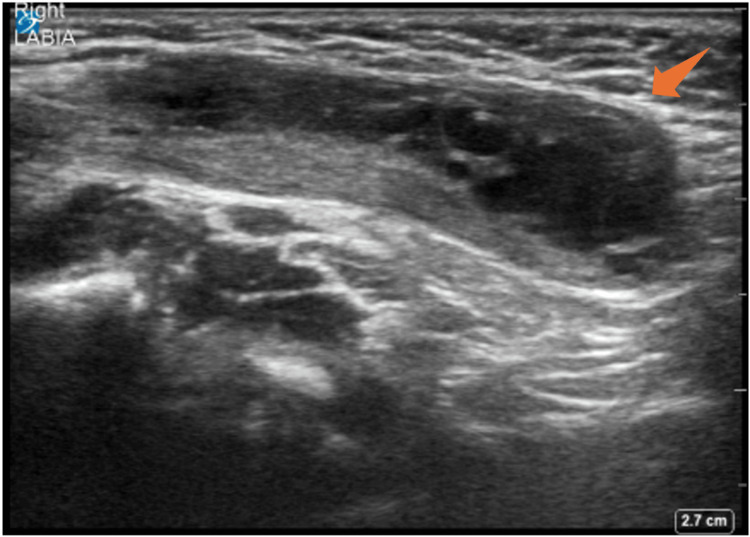
Point-of-care ultrasound of the right groin demonstrating an oval hypoechoic mass with heterogeneous echotexture

**Figure 2:**
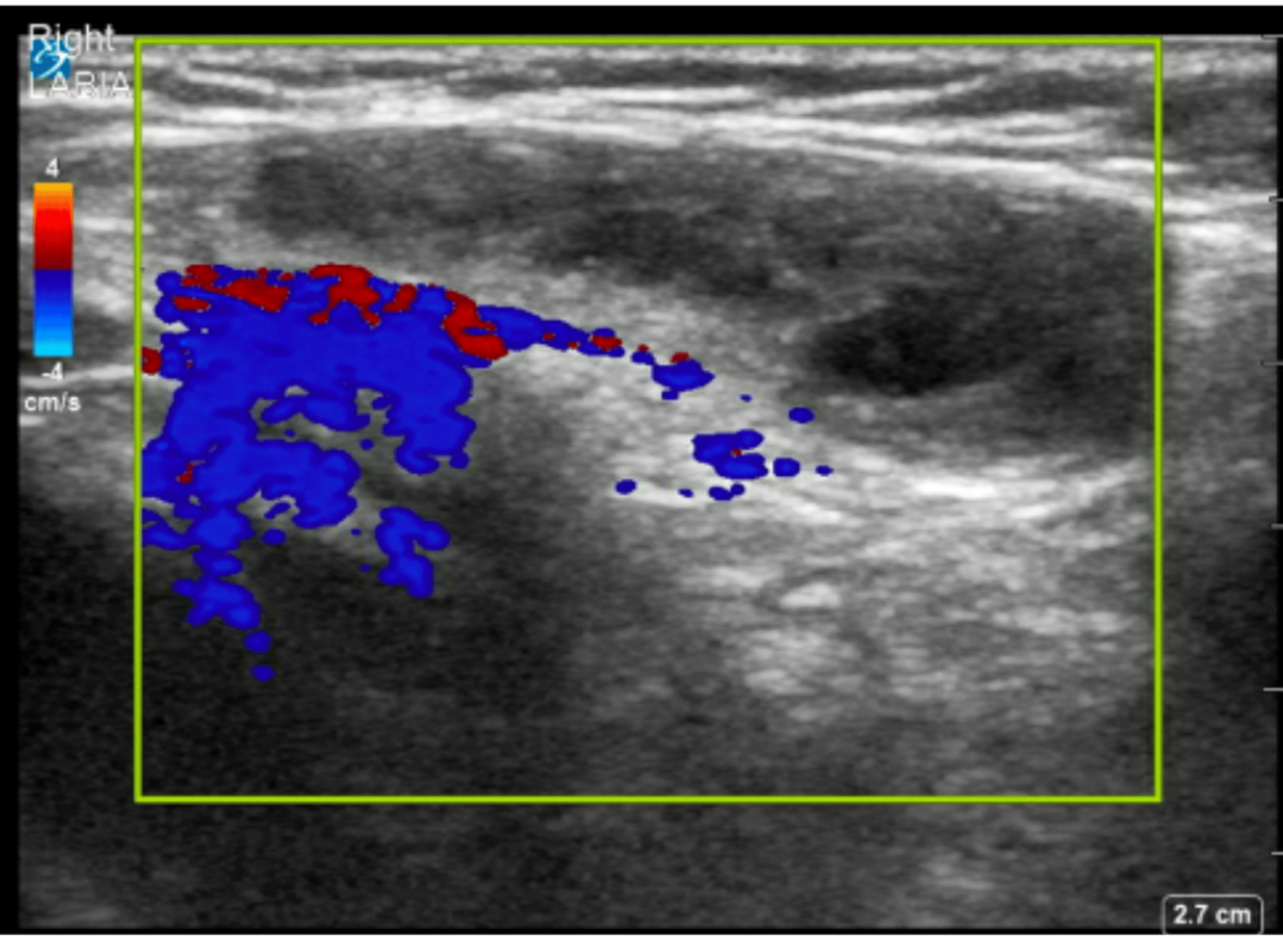
Point-of-care ultrasound of the right groin with color Doppler

A point-of-care ultrasound of the right groin was performed and demonstrated an oval hypoechoic mass with heterogeneous echotexture (Figure 1, Figure 2). A radiology-performed ultrasound showed a 2.4 × 1.0 cm mass with heterogeneous echotexture and internal vascularity, with small follicles, most likely representing the right ovary within the canal of Nuck.

The surgery team was consulted, and upon their evaluation, the ovary had spontaneously reduced. The patient was discharged and advised to follow up with outpatient surgery. She returned to the ED the next day with a recurrent mass in the right inguinal region. Given the recurrence of symptoms within a short interval and the risk of incarceration, the patient was admitted for surgical repair. She underwent bilateral laparoscopic inguinal hernia repair, and the ovary was returned to the peritoneal cavity. She was discharged home the following day.

## Discussion

In females, the rate of ovarian involvement in an inguinal hernia is 15-20% [1,7-9,12-15,17,19]. Ovarian involvement can lead to ischemia, with potential complications including incarceration, strangulation, torsion, and infertility [2,5,10,18-19]. Hernias containing an ovary are associated with a higher risk of incarceration than those containing bowel, necessitating urgent evaluation [1,6-8,12,13].

Ultrasound with color Doppler is recommended as the initial diagnostic tool for pediatric inguinal or pubic masses [4,5,8,9,11,14,16,18]. It is noninvasive, widely available, and avoids radiation exposure [2]. Magnetic resonance imaging may be considered if the ultrasound is inconclusive [5]. When ovarian involvement is suspected, confirming ovarian viability via ultrasound is recommended before attempting manual reduction [4,20]. Sonographic findings suggestive of an ovary include a solid structure containing small peripheral cystic-like structures representing ovarian follicles [8,10,11,16]. Color Doppler should be used to assess ovarian blood flow, and the contralateral side should be evaluated for occult hernias, which are found in up to 88% of cases [1,7,8,11,12,18].

Surgical intervention is indicated in most cases of ovary-containing inguinal hernia; therefore, urgent surgical consultation is essential. Early surgery helps prevent damage to herniated organs [4,8,16,19].

## Conclusions

In female infants with inguinal hernias, the potential presence of an ovary within the hernia must be considered. Compared to hernias containing bowel, ovarian involvement carries a higher risk of incarceration, making prompt assessment and identification critical. When ovarian involvement is suspected, ultrasound confirmation of ovarian viability is recommended before attempting bedside manual reduction. Point-of-care ultrasound is a rapid, safe bedside tool for identifying a herniated ovary. This case report contributes to the growing literature supporting the use of point-of-care ultrasound in the emergency setting to promptly diagnose ovary-containing inguinal hernias, facilitating timely surgical repair and favorable patient outcomes.
